# Risk of Spreading in Adult-onset Dystonia

**DOI:** 10.5334/tohm.952

**Published:** 2024-12-04

**Authors:** Esra Kochan Kizilkilic, Nursena Erener, Mustafa Meric, Nurten Uzun Adatepe, Aysegul Gunduz

**Affiliations:** 1 Istanbul University-Cerrahpasa, Cerrahpasa Medical Faculty, Department of Neurology, Istanbul, Turkey; 2 University of Health Sciences, Prof. Dr. Cemil Taşcıoğlu City Hospital, Department of Neurology, Istanbul, Turkey

**Keywords:** dystonia, focal dystonia, segmental dystonia, spreading, adult-onset dystonia

## Abstract

**Background:**

Adult-onset dystonia can also spread to other parts of the body, although it is not as common as childhood-onset dystonia.

**Objective:**

Our study aimed to examine the clinical factors determining spreading patterns in all adult-onset dystonia types.

**Methods:**

We retrospectively analyzed the medical records of patients with a diagnosis of isolated dystonia followed longitudinally at our center. We included patients reporting symptom onset after 18 years. We then compared the clinical factors between groups with and without spreading.

**Results:**

Among 434 patients (396 focal, 29 segmental, and nine generalized onset dystonia. mean follow-up of 8.6 ± 7.8 years), 48 (11.1%) experienced spread of dystonia, with 37 progressing from focal to segmental, two from focal to generalized, two from segmental to generalized, and seven from focal to segmental to generalized dystonia. Blepharospasm was the most common focal dystonia noted to spread, followed by oromandibular dystonia, cervical dystonia, laryngeal dystonia, and upper extremity dystonia, in decreasing order. A spreading pattern was observed in approximately one in 10 dystonia patients, and the spreading was more frequent in the segmental dystonia group. While there was no difference between the spreading groups regarding sensory tricks, tremor, and gender, family history was more common in the non-spreading group (p = 0.023). Older age at onset was independently associated with increased odds of spreading (hazards ratio: 1.054, p < 0.001, B = 0.053).

**Conclusion:**

Although risk factors for spread are variable, the underlying mechanisms are not fully known. Genetic factors may be possibly related to the spread, and future studies are needed on this subject.

## Introduction

Dystonia, which accounts for approximately 20% of movement disorders, is a hyperkinetic movement disorder characterized by abnormal involuntary movements or posture due to continuous or intermittent muscle contractions [1]. Dystonia is classified according to two main axes: clinical features (axis I) and etiology (axis II) in the 2013 current classification. It is a rather heterogeneous disorder. Clinical descriptors for axis I include: age of onset (infancy, childhood, adolescence, early adulthood, late adulthood), body distribution (focal, segmental multifocal, hemidystonia and generalized), temporal pattern (persistent, action-specific, diurnal fluctuations, paroxysmal), isolated dystonia or combined with another movement disorder and occurrence of other neurological or systemic manifestations. The etiological subcategories for axis II include inherited, acquired, and idiopathic dystonia [2].

Focal dystonia may spread to other body parts and become segmental or generalized. Likewise, segmental-onset dystonia can also become generalized by spreading to a third body region. Although spreading is common in childhood-onset dystonias, it is rare in adult-onset dystonias. Previous studies have investigated the risk factors contributing to the spread of dystonia, including the site of onset, presence of tremor, sensory trick, family history of dystonia, and alcohol sensitivity; however, the data is conflicting [3,4,5].

Studies have shown that blepharospasm spreads much more than other types of focal dystonia [3,6]. Spreading in blepharospasm is also associated with a shorter time from the initial region to the second region than in other dystonias [6]. The site of onset also influences the second body region to which dystonia spreads. Cervical dystonia spreads to the hands, and blepharospasm to the oromandibular and cervical regions [3]. In one study from Italy, the spreading of dystonia to the third body region was investigated. The study found that focal-onset dystonia had a greater trend to transform than patients with segmental/multifocal dystonia at onset [4].

Considering these results, it appears that dystonia subgroups exhibit distinct characteristics. Further investigation and detailed analysis of these features are essential for understanding the biological basis of the disease, determining its prognosis, and identifying improved treatment approaches. In line with these goals, our study aimed to examine the clinical and demographic characteristics of all adult-onset dystonia types, specifically focusing on the spreading dystonia subgroup.

## Methods

### Study design

The local ethics board at Istanbul University-Cerrahpasa approved the study (Date: 16.01.2024, No: 877197). In this retrospective study, the medical records of all patients diagnosed with dystonia who were followed up for an average of 8.6 ± 7.8 years in our Movement Disorders clinic were analyzed. At the time of the assessments, all patients were examined by the same two neurologists (A.G., N.U.A.) using one standardized data sheet.

### Patients

The records of patients who had dystonia without any underlying disorder with symptom onset after the age of 18 years were included in the study. The exclusion criteria were as follows: i) age of dystonia onset <18 years, ii) patients with acquired dystonia due to a specific known cause (vascular causes such as ischemia, hemorrhage, aneurysm, other focal brain lesions, perinatal brain damage, cerebral palsy, parkinsonism, or other neurological disorders, recent use of neuroleptic drugs and toxicity, functional), iii) presence of other neurological comorbidities (such as multiple sclerosis, parkinsonism). Imaging data such as brain and cervical magnetic resonance imaging or computed tomography was used for exclusion.

### Clinical characteristics

The clinical data evaluated included sex, age of onset, age at examination, site of onset, disease duration, if there was spread, the site of spread, how long it took to spread, effective sensory trick, family history of movement disorders, comorbidities and medications used, the areas affected by dystonia, the muscles treated with botulinum toxin injections and the new body parts added, if any, at each follow-up. If there was missing data (such as sensory trick, comorbidities, medications used, and family history of dystonia), we completed it by telephone interviews.

**Figure 1:**
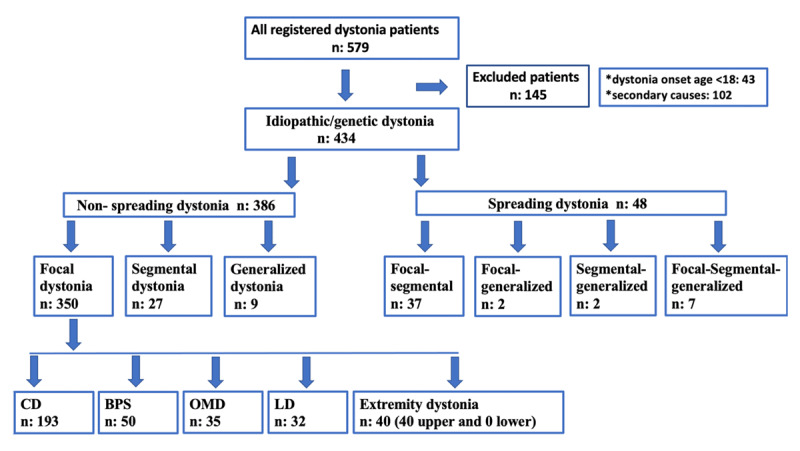
Flowchart of the study. CD: Cervical dystonia; BSP: blepharospasm; OMD: oromandibular dystonia; LD: laryngeal dystonia (LD).

Patients were grouped as focal, segmental, or generalized based on the 2013 classification of dystonia, according to findings at onset and during each examination [2]. Focal, segmental, and generalized dystonia were defined as follows: focal if only one body region was affected; segmental if two or more contiguous body regions were affected; and generalized if the trunk and at least two other body regions were affected. We classified patients into two groups: i. patients with similar patterns of involvement regarding affected body parts (i.e., those with the same endpoint at the onset of dystonia and during the follow-up visits at our center), ii. patients noted to have more body regions affected by dystonia during the follow-up visits. The change of pattern was grouped as focal-segmental, focal-generalized, segmental-generalized, and focal-segmental-generalized, according to the affected body parts at the end of the follow-up period (spreading patterns). The time to spread and body regions of spread were noted based on the patient’s file registration data and botulinum toxin injection procedure data at the follow-up visit. We further categorized patients with focal dystonia according to the site of onset of dystonia: cervical dystonia, blepharospasm, oromandibular dystonia, laryngeal dystonia, and extremity dystonia (hand, arm, leg, foot). Figure 1 shows the study flowchart.

## Statistical analysis

We performed the statistical analysis using the SPSS (Statistical Package for the Social Sciences) for the Windows 20 package program.

First, we compared demographic and clinical findings between patients with similar patterns of involvement regarding affected body parts and patients with different patterns of involvement in the following visits. We used the Chi-square test for categorical comparisons, and for continuous values, we used the Mann-Whitney U test. Then, we compared demographic and clinical findings between patients with different spreading patterns using the Chi-square test for categorical values and the Kruskal-Wallis test for continuous values. We also performed a logistic regression analysis to establish risk factors for spreading patterns in our cohort.

The association of spreading patterns and disease duration was analyzed using multiple logistic regression and Cox-proportional hazard models. The variables entered in the model were sex, age at onset, comorbid disorders (autoimmune diseases, hypertension, diabetes mellitus, etc.), accompanying other movement disorders (myoclonus, parkinsonism, tremor, etc.), sensory trick, and family history.

We also formed Kaplan-Meier curves to analyze the likelihood ratio. We have performed a Kaplan Meier analysis for time to transformation and transformation, including the region involved at onset as the factor.

A p-value <0.05 was considered significant.

## Results

A total of 579 patient files were evaluated retrospectively. We included 434 patients who met the inclusion criteria in the study. A total of 145 patients were excluded from the analysis since 43 had age <18 years at dystonia onset, and 102 patients had dystonia due to structural etiologies.

In the whole group, there were 239 (55.5%) women. The mean age was 59.1 ± 14.7 years. The mean follow-up period was 8.6 ± 7.8 years.

Among 434 patients, 396 had focal-onset, 29 segmental-onset, and nine generalized-onset dystonia. Focal-onset dystonia patients were diagnosed with blepharospasm (n = 65), oromandibular dystonia (n = 44), cervical dystonia (n = 210), laryngeal dystonia (n = 35), and upper extremity dystonia (n = 42). The mean follow-up duration was 8.6 years.

Table 1 shows details of demographic and clinical findings.

**Table 1:** Comparison of groups with and without spreading patterns.


	PATIENTS WITH A SPREADING PATTERN	PATIENTS WITHOUT A SPREADING PATTERN	p

**Sex, F, %**	64.7	54.3	0.177

**Mean age at last admission, y**	58.8 ± 14.8	59.1 ± 14.7	0.969

**Mean age at onset, y**	43.6 ± 14.1	43.1 ± 13.9	0.598

**Accompanying symptoms, %**	51	56	0.775

**Other disorders Autoimmune**	50 11.8	38.8 6.7	0.180

**Sensory trick, %**	50	56	0.444

**Family history, %**	5	19.8	0.023*


*<0.05.

Among all patients, 48 (11.1%) experienced the spreading of dystonia, with 37 progressing from focal to segmental, two from focal to generalized, two from segmental to generalized, and seven from focal to segmental to generalized dystonia. Seven patients were diagnosed with generalized dystonia, which developed gradually over time from focal to segmental and, finally, generalized dystonia. Two patients described the transition from focal directly to generalized dystonia. The percentage of spreading among patients with segmental dystonia (n = 9/27, 25%) was more common than in patients with focal onset (n = 39/350, 10%; p = 0.012). However, seven out of nine patients with segmental dystonia had focal onset.

When we excluded patients with transformation from segmental to generalized dystonia (two patients from segmental to generalized and seven patients from focal to segmental and then generalized), 39 patients with focal onset transformed into segmental or directly generalized dystonia (37 patients focal to segmental and two patients focal to generalize).

Among focal dystonia, the spreading was more common in blepharospasm and oromandibular dystonia (p=< 0.000). The percentage of spread according to dystonia type was as follows: blepharospasm, oromandibular dystonia, cervical dystonia, laryngeal dystonia, and upper extremity dystonia were, in decreasing order, 21.8%, 20.4%, 6.3%, 5.8%, and 2.4%, respectively (Table 2).

**Table 2:** Spreading characteristics in patients with focal dystonia.


	BLEPHAROSPASM (n = 64)	CERVICALDYSTONIA (n = 206)	OROMANDIBULARDYSTONIA (n = 44)	LARYNGEALDYSTONIA (n = 34)	UPPER LIMB DYSTONIA (n = 41)

Spread, n (%)*	14 (21.8%)	13 (6.3%)	9 (20.4%)	2 (5.8%)	1 (2.4%)

**Time to first spread, mean years**	3.9	7.2	4	1.5	1

**The body region of spread**					

Blepharospasm	–	4 (30.7%)	1 (11.1%)	–	–

Cervical	9 (64.2%)	–	8 (88.8%)	2 (100%)	–

Oromandibular	5 (35.7%)	1 (7.6%)	–	–	–

Laryngeal	–	–	1 (11.1%)	–	–

Upper limb	–	6 (46.1%)	–	–	1 (100%)


*The spreading was more common in blepharospasm and oromandibular dystonia (p=< 0.000).

When the region of spread was analyzed, blepharospasm spread most frequently to the neck (64.2%), and oromandibular region (35.7%); cervical dystonia spread most frequently to the upper extremity (46.1%); oromandibular dystonia spread most frequently to the neck (88.8%), and laryngeal dystonia spread most frequently to the neck (100%) (Table 2).

The mean time to first spread was 7.2 years for cervical dystonia, four years for oromandibular dystonia, 3.9 years for blepharospasm, 1.5 years for laryngeal dystonia, and one year for upper extremity dystonia (Table 2).

When comparing patients with and without spread, there were no differences between the groups regarding sex, mean age at the last examination, or mean age at onset. Additionally, accompanying symptoms such as tremor and tremulousness did not differ between the groups. Information regarding sensory tricks was present in 46 patients in the spreading group and 316 in the nonspreading group. There was a sensory trick in 56% of patients with spreading patterns and 50% of non-spreading dystonia (p = 0.444). Although autoimmune disorders such as thyroiditis, allergic asthma, and rheumatological disorders were twice as common in patients with spreading patterns than in patients without spreading patterns, other disorders such as diabetes or hypertension were also common, and the difference was not significant (p = 0.180). Supplementary material 1 presents the autoimmune disorders. Family history was known in 40/48 and 278/386 patients, respectively, and it was significantly more common in patients without spreading patterns than those with spreading patterns (p = 0.023). The family history of the patients included movement disorders such as dystonia, tremor, and Parkinson’s disease.

There were no differences in the mean age of patients with and without spreading patterns. The mean age of dystonia onset in all patients was 43.1 ± 13.9. The mean age of dystonia onset in the spreading group was 43.6 ± 14.1, while it was 43.1 ± 13.9 in the non-spreading group (Table 1). However, there was a trend for a higher median value in the spreading group, which was 46.5 years in comparison to 42 years in the non-spreading group, and regression analysis showed older age at onset was independently associated with increased odds of spreading (hazards ratio: 1.054, p < 0.001, B = 0.053). Regression analysis disclosed no relationship between sex, other disorders, accompanying symptoms, sensory trick, family history, and the spreading pattern in dystonia.

**Figure 2:**
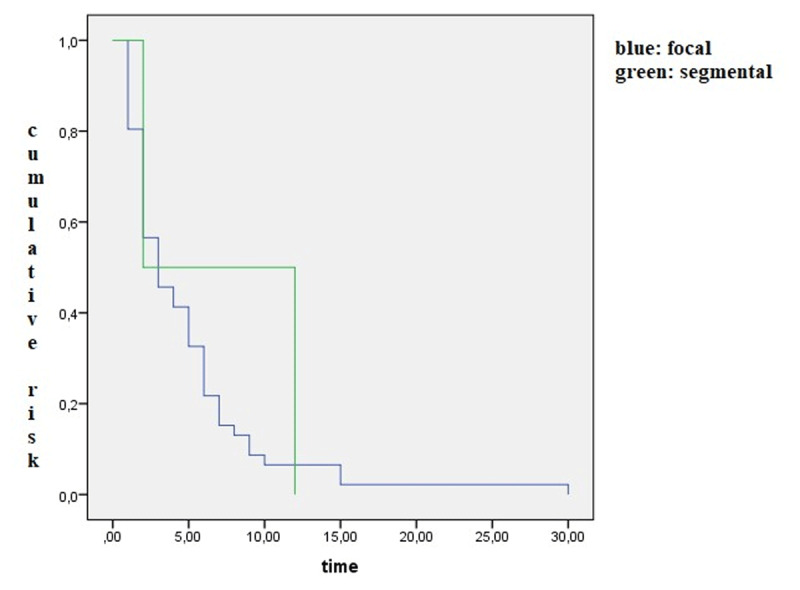
Kaplan-Meier analysis shows the cumulative risk for focal and segmental dystonia over time.

Figure 2 shows the likelihood ratio of spreading in time in focal and segmental dystonia.

## Discussion

The most important findings in this study are as follows: i. almost one out of ten patients with adult-onset dystonia had spreading, ii. there was small evidence that it was more common among segmental-onset patients, blepharospasm and oromandibular dystonia among focal dystonia, and in older patients, and iii. blepharospasm spread more rapidly than cervical dystonia and oromandibular dystonia, with a mean interval of 3.9 years. Among focal dystonia, the spreading was more common in blepharospasm and oromandibular dystonia. Focal dystonia spread to a neighboring segment in most patients; however, some patients with focal onset progressed to generalized dystonia.

Dystonia is the third most common movement disorder after Parkinson’s disease and essential tremor, causing severe physical and psychosocial disability in patients and serious healthcare expenses [7]. The adult-onset focal cervical dystonia is the most common type of dystonia with the highest overall prevalence [7,8]. Blepharospasm is an involuntary spasm of the orbicularis oculi and surrounding muscles. In a previous study, which included 579 adult-onset primary dystonia patients, blepharospasm (329–56.7%) and cervical dystonia (253–43.6%) were the two most common dystonia types, respectively. These were followed by oromandibular dystonia, upper limb dystonia, laryngeal dystonia, and lower limb dystonia [9]. The frequency of different types of dystonia in our study, cervical dystonia and blepharospasm being the two most common focal dystonia types, was consistent with the literature.

The prevalence of spreading (11%) in our study was lower compared to previous studies, in which the transformation rate was between 19.8% and 38% [3,11]. Several factors may account for the lower prevalence. First, the retrospective nature of our study may underestimate the transformation. Second, selecting patients from a treatment cohort may be the second factor. For example, previous studies included only focal-onset dystonia, whereas we also enrolled segmental-onset patients [3,10].

Childhood-onset dystonia is less common but tends to spread and become generalized more frequently. In contrast, adult-onset dystonia is relatively more common and usually remains focal [11]. The spread of dystonia is considered normal in childhood-onset dystonia, where genetic risk factors play an essential role in the pathophysiology. For example, DYT-TOR1 A dystonia usually begins in the leg or arm, and in 60–70% of cases, it spreads to segmental or generalized dystonia over months or years. Almost one out of ten patients with adult-onset dystonia had a transformation from focal to segmental or from focal or segmental into generalized dystonia.

In our study, there was no difference in the mean age of onset of dystonia between the spreading and non-spreading dystonia groups in direct comparisons. However, there was a trend for older age in the spreading group. At the same time, regression analysis revealed a trend, albeit slightly, towards older age in the spreading group, and older age at onset was independently associated with an increased likelihood of spreading. As summarized in supplementary material 2, data regarding the relationship between age at onset and the spread of dystonia in the literature are also contradictory. In a study by Abbruzzese et al. [11], including 124 blepharospasm, 73 cervical dystonia, and 24 focal hand dystonia patients, age at dystonia onset was significantly associated with an increased risk of dissemination. Still, no significant association was found after adjustment for the site of onset [10]. Another study found that older adult-onset patients with blepharospasm had a higher risk of spreading the disease than younger adult-onset patients [12]. In a long-term follow-up study by Stevel et al., older age at dystonia onset was among the most potent predictors of disease spreading; however, the p-value reported by the authors was not significant [13]. In the study conducted by Berman et al. [3], The age at the onset of dystonia was not a risk for the transformation, and another study disclosed that age at the onset did not predict the spread [4]. Although the older age at onset appears to be a risk factor independent of dystonia type in our study, we should acknowledge a bias from the natural course of the dystonia. For example, blepharospasm is more common in elderly patients, and we cannot exclude a relationship between dystonia type and age.

Regarding the region at onset, we showed a small association between spreading and segmental-onset or blepharospasm and oromandibular dystonia among focal-onset patients. A second important difference was the frequency of cervical dystonia, which was far more common among patients included in our study. Blepharospasm, which was more common in a previous study, seems to spread more frequently than cervical dystonia.

In our study and previous studies [11], blepharospasm spread more rapidly than cervical dystonia and oromandibular dystonia, with a mean interval of 3.9 years. In the study by Berman et al., the fastest spreading rate was seen in blepharospasm-onset dystonia at 2.3 years. It was followed by hand (3.1 years), laryngeal (3.3 years) and cervical dystonia (3.5 years), respectively [3]. In a retrospective study by Stevel et al. [13], the mean time to disease progression was longer for patients with torticollis, blepharospasm, and hand dystonia (6 to 15 years) [13]. In our study, upper extremity dystonia and laryngeal dystonia spread the fastest. However, we cannot distinguish whether this bias was related to their lower prevalence than other groups.

Several studies have been published on the spreading and risk factors for spreading adult-onset focal dystonias. However, literature data on the spread of segmental dystonia are limited. Literature reported more frequent spreading in focal onset than segmental/multifocal dystonia [4].

Studies on this subject have found a higher transformation rate in dystonia presenting with blepharospasm, while cervical dystonia has been associated with a low rate of spreading [5,6,14]. In a study by Martino et al. [5], patients presenting with blepharospasm had a two-fold higher spreading rate than cervical dystonia [5]. In another study, the group with the highest probability of further spread was observed in patients with blepharospasm at onset (33.3%), followed by upper limb dystonia (32.3%), torticollis (19.6%) and laryngeal dystonia (6.7%). In this study, blepharospasm was also associated with the highest rate of spread, with the second site affected after an average of 1.2 years [6]. According to Berman et al., the spreading prevalence was in 8.4% of patients with neck onset, and 50% of patients with cranial onset has been reported [3]. The prospective cohort conducted in 2020 [3] found that patients with blepharospasm most spread to the oromandibular region and neck. However, in clinical practice, generalization was uncommon, whereas our study also describes a more generalized pattern in patients with either segmental or focal onset. Generalization after focal onset was more possible if it first transformed to segmental from focal. In contrast, in Bergman’s study, patients with cervical dystonia most frequently spread to the hand, and those with hand or laryngeal dystonia most spread to the neck [3]. We suggest that these patients may have a predisposing factor. Although autoimmunity was indicated as a risk factor in some of the dystonia syndromes [15], the accompanying autoimmune disorders were not different between groups in our study. Could genetic factors play a determining role? In our opinion, this is a question to be answered.

Interestingly, our study observed a lower probability of transformation in patients with a positive family history, which was compatible with the prospective study by Stevel et al. [6]. The results regarding the relationship between family history and spread are controversial in the literature. While some studies [3] support the role of family history in increasing the risk of spread, other studies [16] did not find a significant association between the spread of adult-onset dystonia and family history. Therefore, despite the negative findings related to age and family history, we suggest that genetics may still play a role in a subgroup of patients with generalized dystonia, where the transformation from adult-onset segmental dystonia to generalized dystonia occurred. To us, this subject may still warrant investigation.

Tremor was supposed to be a risk factor for spread in one study [17], but other studies have failed to replicate such a relationship [3,6]. Similarly, we could not reveal an association between the presence of tremor or tremulousness and the transformation of dystonia. The results of the relationship between the presence of a sensory trick and the risk of dystonia spread are also contradictory in the literature. In our study, there was no significant difference between the groups regarding the presence of sensory tricks.

We should acknowledge the limitations of the study. First, the retrospective design and retrospective analysis of the medical files may compromise the accuracy of the information about the clinical findings of dystonia, such as spreading pattern, age at dystonia onset, and time of transformation. However, all patients were examined by the same physician using the same datasheet at each follow-up. The patient population is large, although all patients were recruited from one center. Because these patients were administered botulinum toxin, the examination of each segment was detailed and included electrophysiology if indicated by the primary physician. Although the total population was large, the sample size was small in certain types of dystonia, such as lower extremity dystonia or segmental onset dystonia. However, this was secondary to natural history. Another limitation of our study is that no retrospective review was done regarding the time/duration/dose of botulinum neurotoxin applied to the patients, and its distribution relationship was not evaluated. The uneven numbers of spreading and non-spreading groups in the statistical analysis may bear a bias. However, this was due to the frequency of spreading in the natural history of adult-onset dystonia. Lastly, selection bias may have occurred because our cases were identified from a tertiary referral movement disorder center.

In conclusion, nearly 10% of adult-onset dystonia are at risk of transformation. Older age is a risk factor for generalization. However, even focal dystonia may spread and become generalized. No clinical finding predicted transformation. Our future prospect is to analyze different genetic factors to determine a potential biomarker to predict transformation.

## Data Accessibility Statement

The data supporting this study’s findings are available from the corresponding author upon reasonable request.

## Additional Files

The additional files for this article can be found as follows:

10.5334/tohm.952.s1 Supplementary material 1. The frequencies of autoimmune disorders in patients with and without spreading pattern.

10.5334/tohm.952.s2 Supplementary material 2. Review of risk factors for transformation in dystonia.
