# Supplementary material for: Risk of Spreading in Adult-onset Dystonia
Source: Tremor Other Hyperkinet Mov (N Y). 2024 Dec 4;14:59. doi: 10.5334/tohm.952 (PMC11623075; doi:10.5334/tohm.952)
Supplement: Supplementary material 1. — The frequencies of autoimmune disorders in patients with and without spreading pattern. [file tohm-14-1-952-s1.pdf]

Supplementary Material 1. The frequencies of autoimmune disorders in patients with and without spreading pattern

	**Patients with a spreading pattern n= 34***	**Patients without a spreading pattern n=323***	**p**
Hashimoto's thyroiditis	4 (11.77%)	7 (2.1)	0.180
Psoriasis	0	1 (0.3%)	
Familial Mediterranean Fever	1 (3%)	1(0.3%)	
Diabetes mellitus	2 (2.88%)	18 (5.5%)	
Hypertension	11 (32.35%)	50 (15.4%)	

* Number of dystonia patients with known comorbidities
