# Supplementary material for: Risk of Spreading in Adult-onset Dystonia
Source: Tremor Other Hyperkinet Mov (N Y). 2024 Dec 4;14:59. doi: 10.5334/tohm.952 (PMC11623075; doi:10.5334/tohm.952)
Supplement: Supplementary material 2. — Review of risk factors for transformation in dystonia. [file tohm-14-1-952-s2.pdf]

Supplementary material 2. Review of risk factors for transformation in dystonia.

	**Types of dystonia evaluated**	**Age at onset**	**Sex**	**Family history**	**Sensory trick**	**Tremor**	**Type of dystonia**
**Defazio., 1999**	BPS	A	A	Non-a	Non-a	Non-a	NA
**Weiss., 2006**	CD, BPS, LD, OMD, ULD, LLD	A	NA	NA	NA	NA	A*
**Svetel., 2007**	CD, BPS, LD, ULD	A	Non-a	A	NA	NA	A*
**Abbruzzese., 2007**	CD, BPS, ULD	A	Non-a	NA	NA	Non-a	A*
**Martino., 2012**	CD, BPS	A	NA	NA	NA	NA	A*
**Stevel., 2015**	CD, BPS, LD, ULD	A	NA	A	NA	A	A*
**Berman., 2020**	CD, BPS, LD, ULD	A	Non-a	A	Non-a	Non-a	A*
**Ercoli., 2021**	CD, BPS, LD, OMD, ULD	Non-a	Non-a	Non-a	Non-a	Non-a	Non-a
**Our study, 2024**	CD, BPS, LD, OMD, ULD	A	Non-a	A	Non-a	Non-a	A*

A: Associated, **NA**: Not Available, **Non-a:** Not associated, **A*:** Associated with blepharospasm

CD: Cervical Dystonia, **OMD**: Oromandibular Dystonia, **LD:** Laryngeal Dystonia, **BPS:** Blepharospasm, **ULD:** Upper Limb Dystonia, **LLD:** Lower Limb Dystonia
